# Effect of Nano Ferrochrome Slag-Infused Polymer Matrix on Mechanical Properties of Bidirectional Carbon Fiber-Reinforced Polymer Composite

**DOI:** 10.3390/polym17182527

**Published:** 2025-09-18

**Authors:** Sultan Saif Al mamari, Sabariah Julai, Mohd Faizul Mohd Sabri, Lenin Anselm Wilson Annamal, S. M. Shahabaz

**Affiliations:** 1 Department of Mechanical Engineering, Faculty of Engineering, Universiti Malaya, Kuala Lumpur 50603, Malaysia; faizul@um.edu.my; 2 Engineering Department, University of Technology and Applied Sciences, P.O. Box 77, Shinas PC 324, Oman; lenin.wilson@utas.edu.om; 3 Department of Mechanical Engineering, P.A. College of Engineering, Affiliated to Visvesvaraya Technological University, Belgavi 574153, Karnataka, India; shahbaaz.mech@pace.edu.in

**Keywords:** carbon fiber reinforced polymer (CFRP), ferrochrome slag, nanofillers, mechanical properties, scanning electron microscope (SEM)

## Abstract

Polymeric nanocomposites have been extensively investigated due to their potential for enhancing the mechanical and tribological properties of polymer composites. In this study, the mechanical performance of carbon fiber-reinforced epoxy composites modified with nano-sized ferrochrome slag particles, an industrial by-product from stainless steel manufacturing, was evaluated. Composite laminates were fabricated using a vacuum-assisted hand lay-up process, with consistent carbon fiber reinforcement and uniformly dispersed nanofillers in the epoxy matrix. Mechanical properties such as tensile, flexural, impact, and Shore D hardness were evaluated as per ASTM and ISO standards. At 2 wt.% nanofiller loading, enhanced tensile strength and hardness by 33.02% and 8.92%, respectively, were achieved, while flexural strength and impact strength increased by 3.70% and 3.62% at 1 wt.% compared to the neat composite. Higher filler contents (>3 wt.%) resulted in reduced performance due to particle agglomeration and microstructural inhomogeneity. A scanning electron microscope was used to determine the uniform dispersion and agglomeration of nanofillers. The results demonstrated the potential of ferrochrome slag as a sustainable and cost-effective nanofiller for advanced composite applications.

## 1. Introduction

Composite materials have gained significant importance in advanced engineering applications due to their superior mechanical properties and design flexibility. Among these, carbon fiber-reinforced polymers (CFRPs) stand out as high-performance materials characterized by an excellent strength-to-weight ratio, high stiffness, fatigue resistance, and resistance to environmental degradation [1]. These advantages have made CFRPs a preferred choice in demanding sectors such as aerospace, automotive, marine, wind energy, and civil infrastructure [2]. However, the performance of CFRPs is critically dependent on the matrix phase—typically an epoxy resin—which binds the fibers and transfers loads. Despite its advantages, epoxy resin is inherently brittle, thermally insulating, and vulnerable to crack propagation, which compromises the overall toughness and long-term reliability of CFRP systems [3]. To address these challenges, researchers have focused on modifying the epoxy matrix with various micro- and nanoscale fillers. Incorporating nanofillers into the matrix has proven to improve interfacial bonding, mechanical strength, impact resistance, and thermal conductivity. Fillers such as carbon nanotubes (CNTs) [4], silica (SiO_2_) [5], alumina (Al_2_O_3_) [6], graphene [7], and silicon carbide (SiC) [2] have shown promise in enhancing the functional properties of epoxy matrices. Kiran et al. reported that the introduction of CNTs into epoxy–carbon composites resulted in improved tribological behavior and increased wear resistance. These enhancements were further optimized using a machine learning approach to determine ideal resin-to-filler ratios and filler arrangements [8]. Similarly, Shcherbakov et al. observed an 11% increase in tensile strength by adding 0.05 wt part of CNT filler compared to unfilled composite [9]. Bunea observed that the addition of graphene to epoxy–carbon composites increased tensile strength, while the combination of graphene with aramid powder and other fillers provided higher impact resistance [10]. Rashid et al. found that SiC fillers improved both composite strength and thermal conductivity, making them suitable for applications requiring heat dissipation alongside structural performance [11]. Ba-Gubair et al. confirmed that carbon fiber-reinforced composites containing alumina nanofillers exhibited improved physical properties, specifically strength and durability [12]. Similarly, Bodduru et al. noted that the composites containing CNT-MXene hybrids showed enhanced vibration and damping properties [13]. Hybrid nanofillers, including nano-hydroxyapatite (nano-HAP), were reported to reduce wear volume and improve adhesion strength by Somashekar et al., thereby enhancing tribological performance [14]. Amal et al. observed that if a composite has hybrid fillers (multiple fillers) instead of a singular filler system, impact resistance was increased [15]. Also, the fracture energy during impact seemed to be dependent on the sequence and arrangement of the fillers. In addition, ferrochrome slag is also used as filler in cementitious systems with improved mechanical properties. Hang et al. noted that incorporating 15% of ferrochrome slag powder showed an increase in compressive strength up to 15.8% [16]. Al-Jabri et al. observed crucial improvement in the mechanical properties, including compressive strength (33%) and flexural strength (39%) of mortars by replacing sand with ferrochrome slag at 20% [17]. Reddy and Konda observed an enhancement in compressive, flexural, and tensile strength by replacement of aggregate (granular materials like sand, gravel, stone, etc.) with ferrochrome slag at 75% filler addition [18]. Table 1 provides an overview of the effects of different fillers on the mechanical nature of epoxy–carbon fiber composites in order to show their performance in terms of tensile strength, impact resistance, and wear behavior, among other important characteristics.

Despite the benefits of these advanced fillers, they often suffer from high production costs, complex processing requirements, limited availability, and potential environmental and health hazards during handling. Therefore, there is growing interest in identifying cost-effective, sustainable, and environmentally benign alternatives that can be incorporated into polymer composites without compromising performance. To overcome these limitations, one of the alternatives is the utilization of industrial waste materials as reinforcement fillers, such as industrial by-products like fly ash, red mud, and metallurgical slags, which have been explored in recent years for their potential to enhance composite properties while addressing issues of waste disposal. Ferrochrome slag, a by-product of ferrochrome alloy production in stainless steel manufacturing, is rich in hard ceramic oxides such as silica (SiO_2_), alumina (Al_2_O_3_), and magnesia (MgO) [23]. These ceramic phases possess high hardness, thermal stability, and chemical resistance, making ferrochrome slag a suitable candidate for use as a reinforcing filler in polymer matrices. Repurposing this slag not only reduces the environmental footprint associated with its disposal but also contributes to sustainable material development aligned with circular economy principles [24]. The slag composition typically contains SiO_2_ (30–40%), making it hard and thermally resistant [25]. And some of the other significant materials include alumina (15–25%), which enhances the heat resistance and wear properties, and MgO (8–12%), which improves thermal stability along with durability [26,27]. The chromium oxide (Cr_2_O_3_), present in trace quantities (5–10%), gives the material its anti-oxidation and corrosion properties [28]. Other constituents, such as calcium oxide (CaO) and trace oxides, increase the melting points of the ferrochrome, making it much more inert chemically, while iron oxide (FeO) contributes to the mechanical strength of the slag, without leaching during the dispersion in the polymer [29].

In addition to its chemical composition, the physical characteristics of ferrochrome slag make it an appealing filler for epoxy–CFRP systems. The material has a density in the range of 2.7–3.0 g/cm^3^, which is suitable for structural applications. Its crystalline oxide structure gives it high hardness and oxidation resistance, thereby enhancing wear performance in composites [30,31]. Ferrochrome slag typically ranges from 10 to 150 µm, although smaller particles are preferred to ensure uniform dispersion in the epoxy matrix. Its thermal stability is also high since it has a melting point of magnitude greater than 1600 °C. Furthermore, post-treatment processes can reduce the porosity, thereby improving the mechanical reinforcement properties as well as the compatibility with the resin systems [32]. These characteristics make ferrochrome slag a robust and versatile filler for advanced composite applications, as ferrochrome slag presents a more economical option compared to conventional high-performance nanofillers like CNTs, alumina, or SiC, while still enhancing composite properties [33,34].

Despite its potential, the application of ferrochrome slag as a nanofiller in high-performance composites remains relatively underexplored. Most studies to date have focused on micro-sized particles or bulk slag in construction and engineering materials. The potential benefits of using nano-sized ferrochrome slag particles in CFRP composites—such as improved load transfer, enhanced crack resistance, and increased surface hardness—have not been widely reported. While ferrochrome slag has been utilized in construction materials as micro-sized particles, this study is the first to investigate its potential as a nanofiller in advanced composite systems. The study evaluates the influence of varying nanofiller loadings (ranging from 0% to 5% by weight) on tensile, flexural, and impact strength, along with the Shore D hardness. The primary objective is to identify an optimal filler concentration that maximizes performance without inducing adverse effects such as embrittlement or particle agglomeration. In the following section, the methodology followed for the research is explained.

## 2. Materials and Methods

### 2.1. Materials

Carbon fiber-reinforced epoxy nanocomposites were prepared using woven carbon fiber as the reinforcement and epoxy resin (West System 105) as the polymer matrix. The density and diameter of the carbon fiber measured were 2 g/cm^3^ and 7 µm. The resin consists of a liquid blend of both bisphenol A and bisphenol F containing reactive epoxy groups primarily from glycidyl ethers. The curing agent (hardener) used was West System 206, consisting of a blend of aliphatic amines and aliphatic amine adducts based on diethylenetriamine and triethylenetetramine, which react with these epoxy groups to form a crosslinked network [35]. The epoxy was mixed with a slow hardener in a 5:1 ratio by volume, producing a low-viscosity system selected for its extended working time and high cured strength. The reinforcement was maintained at 50 wt.% in all samples. Six composite variants were fabricated with nominal nanofiller contents of 0, 1, 2, 3, 4, and 5 wt.% of the total composite weight, as shown in Table 2.

### 2.2. Preparation of Nanofillers

**Figure 1:**
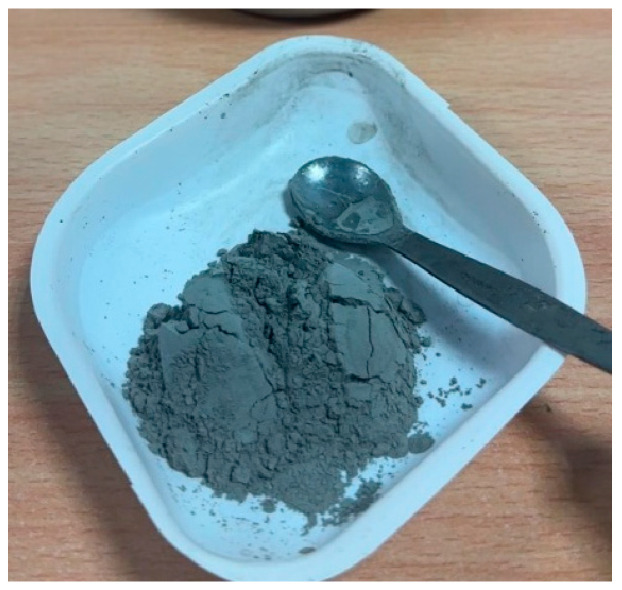
Ferrochrome slag powder after the two-step process.

Ferrochrome slag waste, as shown in Figure 1, was used as the raw material to produce nano-sized particles, which were collected from Gulf Alloys and Metals LLC, Oman. Ferrochrome slag was subjected to a number of processes to reduce the size (in nanometers). A two-step process was used for preparation. Firstly, the amount of ferrochrome slag was ground into microscale particles using a Twin Screw Extruder Machine (70–100 microns), then the ball milling machine was used to obtain ferrochrome slag nanofillers. The overall cost for the preparation of 1 kg of nanofiller was 80 USD, which is lower compared to the other nanofillers, such as Alumina and SiC, which cost around 360 to 400 USD per kilogram. Dry milling was accomplished with no surfactants added during the process. The parameters used for the dry milling process are represented in Table 3.

### 2.3. Composite Fabrication

**Figure 2:**
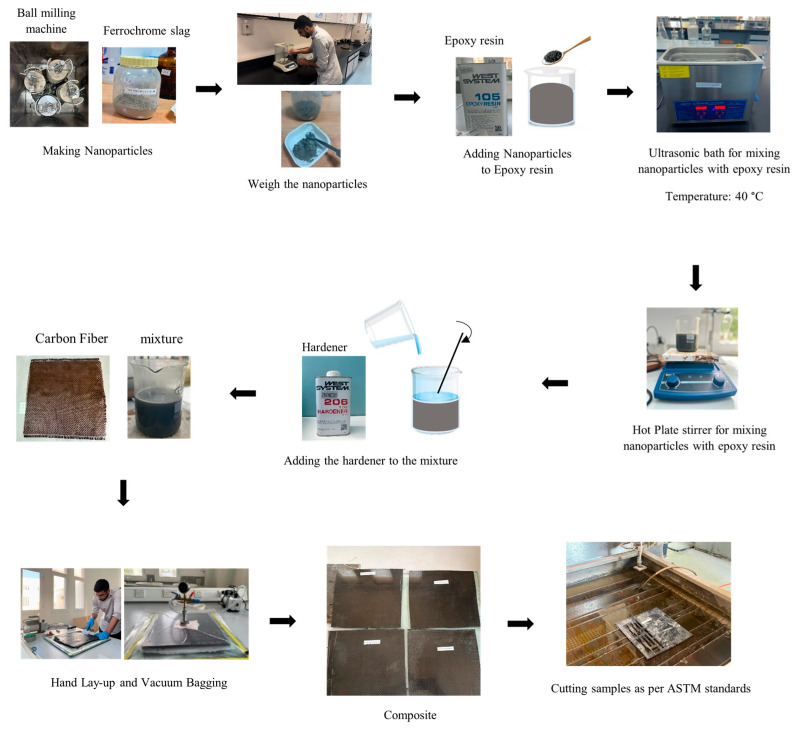
Flowchart of the fabrication process of the composite.

A hand lay-up followed by a vacuum bagging technique carried out at 0.8 bar pressure for a duration of 30 min was employed to fabricate the carbon fiber-reinforced laminates, as shown in Figure 2. The fabricated composites were cured at room temperature (25 °C), and the laminates were demolded and post-cured again at ambient conditions (25 °C) for a duration of 24 h to reach full polymer crosslinking and achieve maximum properties. For the preparation of the nanofilled composites, the measured quantity of ferrochrome slag nanopowder (corresponding to the desired wt.% of the total composite) was added to the appropriate amount of epoxy resin and initially hand-stirred to wet the particles. Then, the nanofillers were gradually introduced into the epoxy resin, where the resin–nanofiller mixture was placed in an ultrasonic bath at 40 °C for 1 h to break up particle agglomerates and achieve a uniform dispersion. Simultaneously, a magnetic hot-plate stirrer was used for an additional 1 h of mixing to ensure homogeneous distribution of the nanofillers within the epoxy. The nanofiller solution was mixed with the hardener in a ratio of 5:1. The mixing and degassing were performed to minimize air entrapment. The thickness of the cured composite panels achieved was 3 to 4 mm. Following fabrication, the test specimens were cut using a water-cooled diamond blade cutter (water-jet cutting) machine.

### 2.4. Mechanical Testing

Specimens were cut according to the required ASTM and ISO standards for each mechanical test. Before testing all samples, edges were polished to remove any notches or roughness from cutting. Five specimens were prepared for each mechanical test, and their average values with standard deviations were recorded. Mechanical tests were conducted to evaluate tensile, flexural, impact strength, and hardness of the composite.

#### 2.4.1. Tensile Test (ASTM D638)

**Figure 3:**
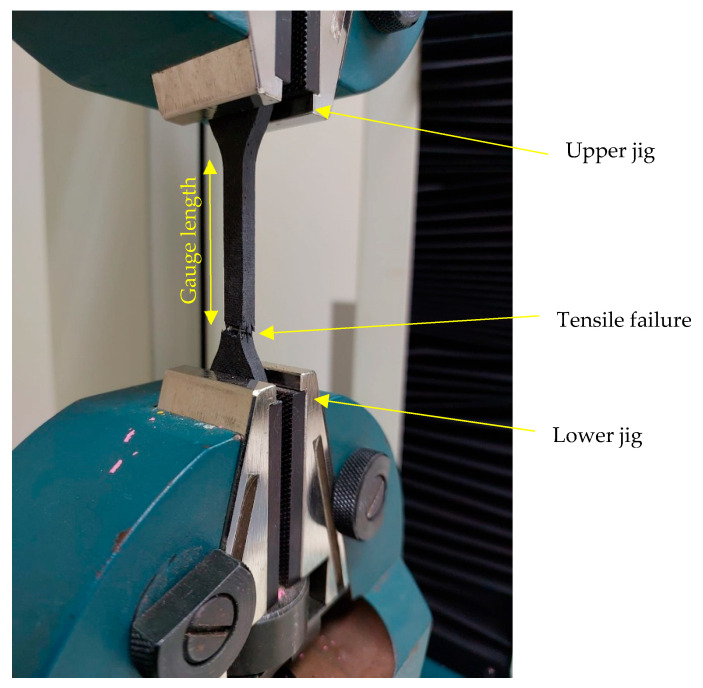
Tensile testing experimental setup.

**Figure 4:**
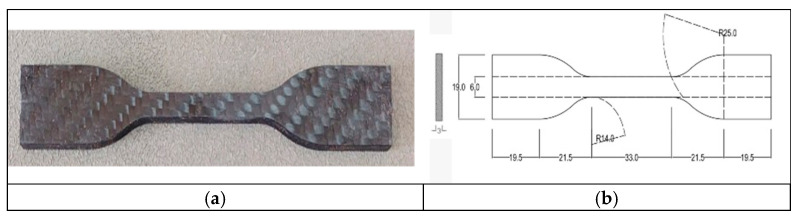
Tensile specimen: (**a**) dog bone shape specimen; (**b**) dimensions of tensile specimen as per ASTM D368.

Tensile properties were evaluated using an Instron Universal Testing Machine (UTM), and testing was performed at ambient room temperature. Bone-shaped specimens (115 × 3 mm) were loaded at a quasi-static crosshead speed until failure [36]. Figure 3 represents the experimental setup of tensile testing. Figure 4 shows the specimen and dimensions of the specimen used for tensile testing. The load was recorded by a load cell, and tensile strength (ultimate stress) was calculated based on the specimen cross-sectional area. Strain was monitored with an extensometer over the gauge length.

#### 2.4.2. Flexural Test (ASTM D790)

**Figure 5:**
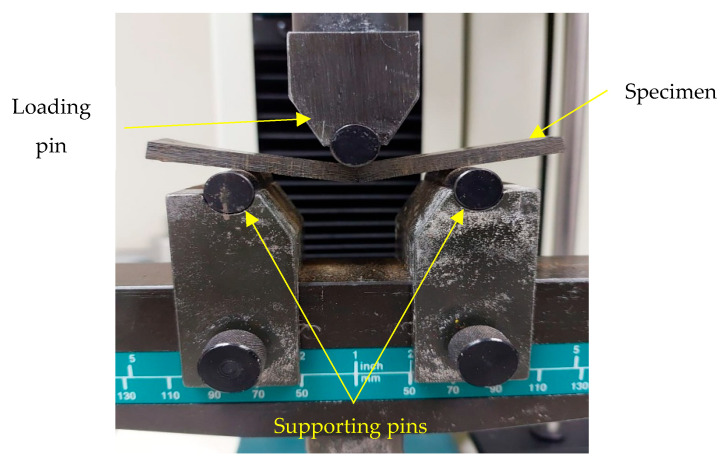
Flexural testing experimental setup.

**Figure 6:**
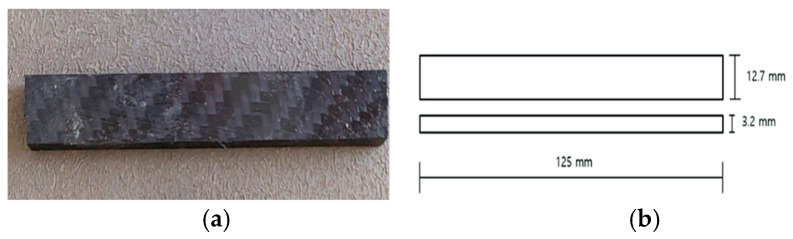
Flexural specimen: (**a**) flexural test specimen; (**b**) dimensions of flexural specimen as per ASTM D790.

A three-point bending test was performed on rectangular bar specimens with dimensions of length (125 mm), width (12.7 mm), and thickness (3.2 mm), following a length-to-thickness ratio of 16:1, using the UTM with a bending fixture, as shown in Figure 5. The span length between two supporting pins was maintained at 51.2 mm with a load applied at the midpoint [37]. The dimensions of the specimen are represented in Figure 6. The flexural strength was determined from the maximum stress at failure (at 5% strain) using the standard formula. All flexural tests were carried out until the specimens fractured or showed a load drop indicating failure.

#### 2.4.3. Hardness Test (Shore D, ASTM D2240)

**Figure 7:**
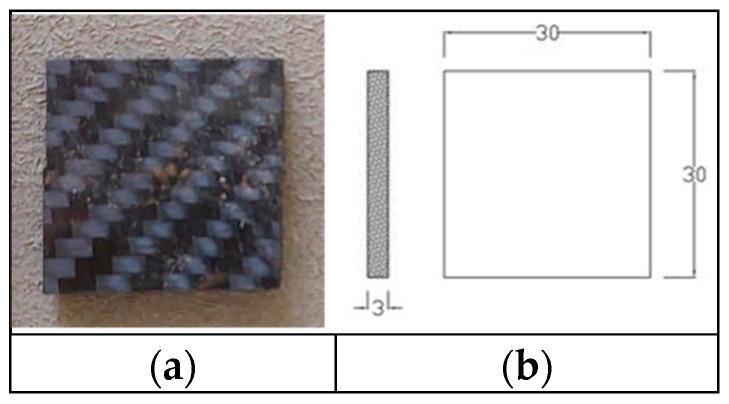
Hardness specimen: (**a**) hardness testing specimen; (**b**) hardness specimenwith dimensions.

Surface hardness was measured using a Shore D durometer (SLX-D Shore Durometer) following ASTM D2240 [38]. The durometer indenter was pressed into flat, smooth regions of the composite, and the hardness reading (dimensionless Shore D scale 0–100) was recorded once stabilized (1–2 s). Figure 7a,b represents the hardness specimen. Ten measurements were taken at different locations on each sample and averaged to obtain a representative hardness value. The standard deviation of the readings was computed to assess variability.

#### 2.4.4. Impact Test (ISO 179)

**Figure 8:**
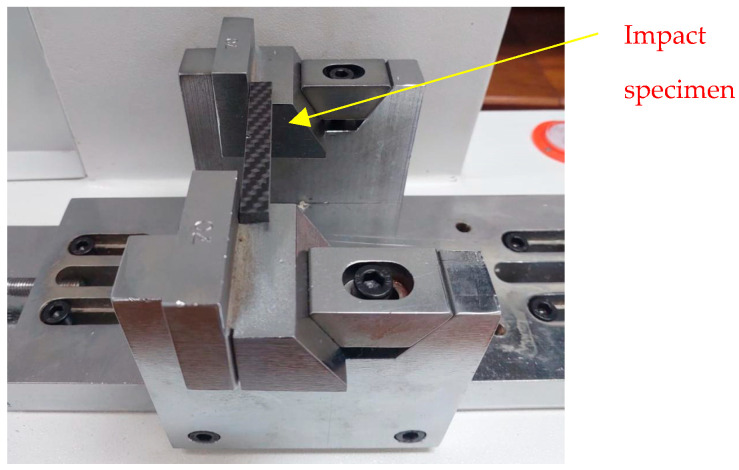
Impact testing experimental setup.

**Figure 9:**
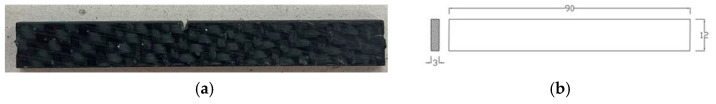
Impact specimen: (**a**) impact test specimen; (**b**) dimensions of impact specimen as per ISO 179.

The Charpy impact test was performed based on the ISO 179 standard, which measures the impact strength of specimens [39]. Figure 8 represents the experimental setup of the Charpy impact test performed. The test was performed using the NL Scientific universal impact tester (TT 6002 X/005). A ‘Type B’ notch specimen was used for impact testing that was supported horizontally, where the pendulum head with a pendulum energy of 25 joules was dropped from a specific height. As it impacts the specimen, the energy absorbed is measured. The energy absorbed by the specimen during fracture indicates its resistance to sudden impact. The dimensions of the specimen as per the standard are 90 × 12 × 3 mm, as shown in Figure 9.

## 3. Results and Discussion

All mechanical tests were conducted in the laboratory at ambient temperature conditions (25 ± 2 °C), and the test equipment was calibrated before use. The following section presents the results of these tests along with an analysis of the effect of nanofiller content on the composite’s mechanical performance.

### 3.1. SEM Analysis

**Figure 10:**
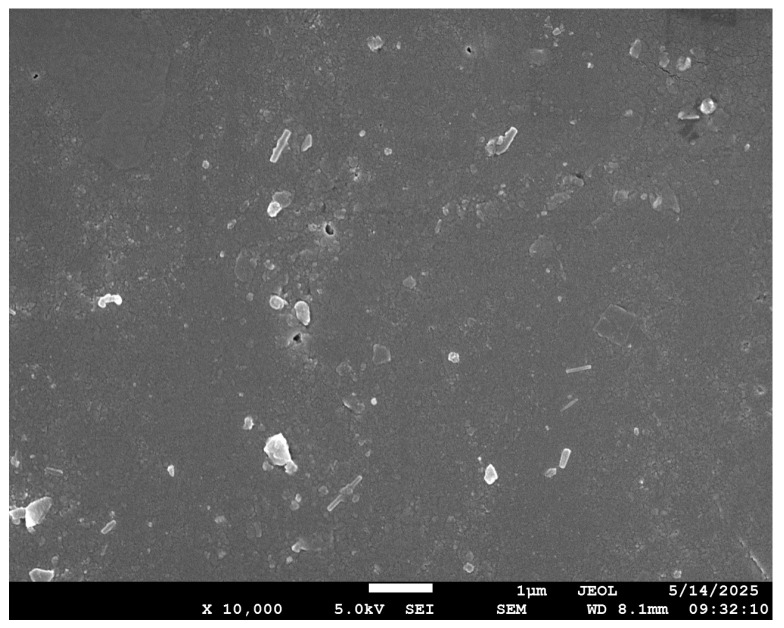
Particle size of ferrochrome slag nanofiller.

**Figure 11:**
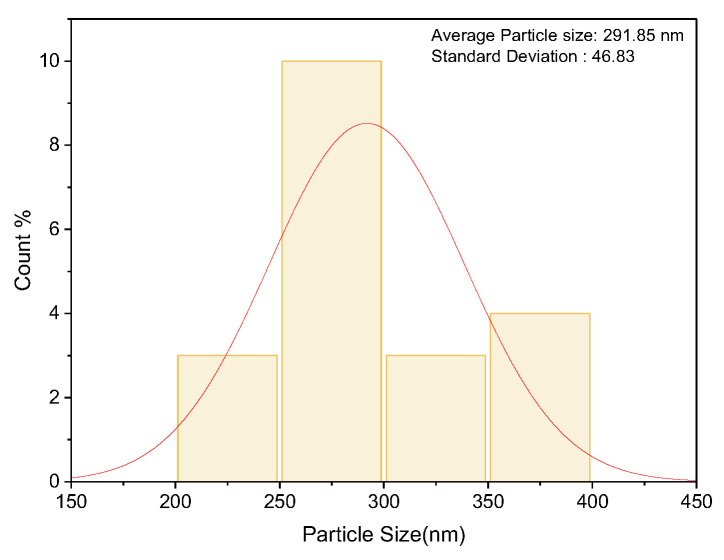
Histogram of particle size distribution of ferrochrome slag nanofiller.

Scanning Electron Microscopy (SEM) was performed to confirm the uniform dispersion of nanofillers and analyze the agglomeration of nanofillers at higher filler loading. Achieving uniform nanofiller dispersion within the epoxy matrix is a key challenge, as it can greatly influence the mechanical performance of the final composite. When dispersed effectively, nanofillers can hinder crack propagation, improve stress distribution, and reinforce the matrix without altering the fundamental architecture of the fiber-reinforced structure. SEM analysis (Figure 10) was performed to determine the nanofiller size. The average particle size obtained was 291.85 nm. The histogram (Figure 11) for the same was plotted to show the distribution of particle size, and the average size and the standard deviation of the particle size in detail are represented in Table 4.

**Figure 12:**
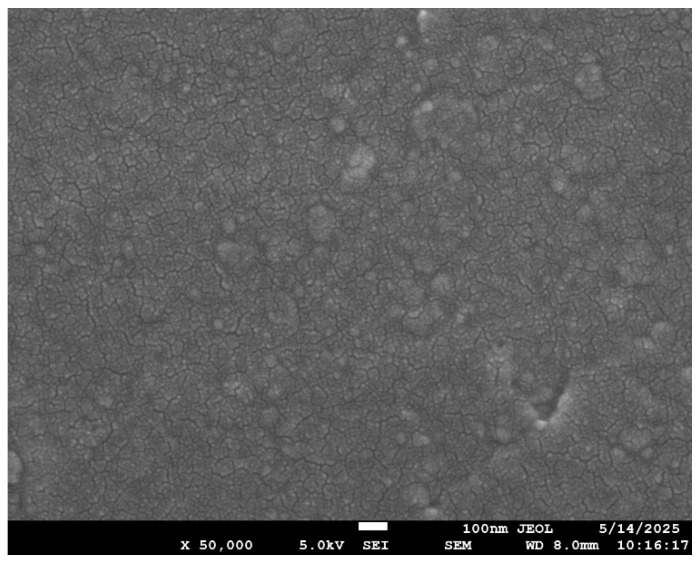
SEM image of neat CFRP without any filler.

**Figure 13:**
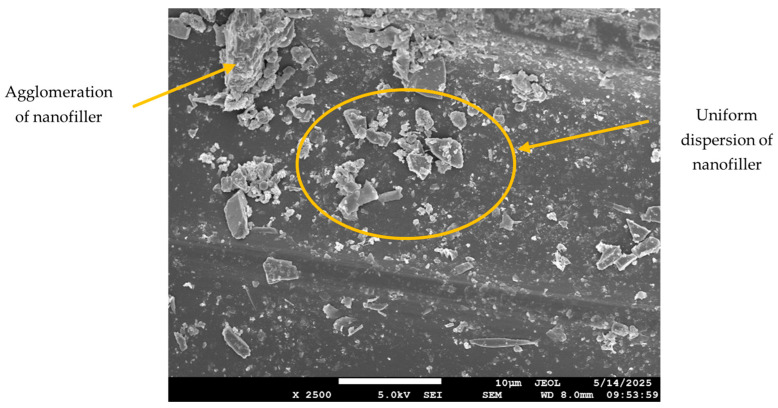
Dispersion of ferrochrome slag nanofiller at a filler loading of 2 wt.%.

**Figure 14:**
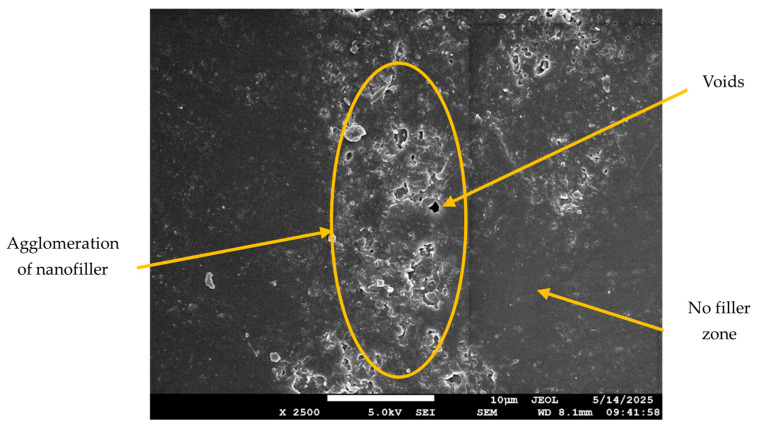
Dispersion of ferrochrome slag nanofiller at a filler loading of 5 wt.%.

**Figure 15:**
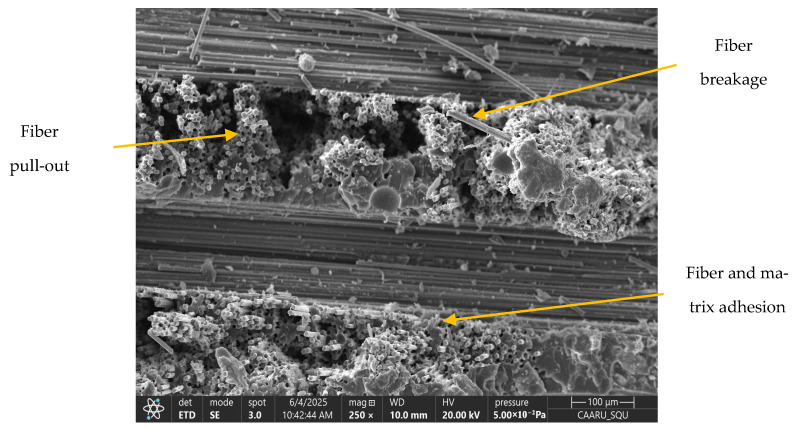
SEM image of failure specimen.

The SEM images for the neat composite (no filler) and those with filler loadings of 2 wt.% and 5 wt.% are shown in Figure 12, Figure 13, Figure 14 and Figure 15. At 2 wt.% filler loading (Figure 13), the nanofillers appear uniformly dispersed with minimal agglomeration, indicating the effectiveness of the combined ultrasonication and mechanical stirring process. When the filler loading increases to 5 wt.%, pronounced agglomeration and surface voids are observed. This occurs due to the high surface area of the nanofillers, which promotes strong attractive Van der Waals forces, leading to extensive cluster formation within the composite. The non-uniform dispersion at higher filler loadings also results in filler-free zones, as seen in Figure 14. Although some agglomeration of nanofillers is visible at 2 wt.% filler loading, the extent of agglomeration is significantly higher at increased filler additions. These microstructural irregularities reduce the overall performance of the composite, ultimately causing premature failure, which is explained in further sections. Figure 15 represents the SEM image of the failure specimen. From Figure 15, it can be observed that the nanofillers have been uniformly distributed, and a strong fiber–matrix adhesion can be noted, which further indicates better bonding and wetting of the nanofillers with epoxy resin. Some of the failure mechanisms that are observed from the figure are fiber breakage and fiber pull-out.

### 3.2. EDS Analysis

**Figure 16:**
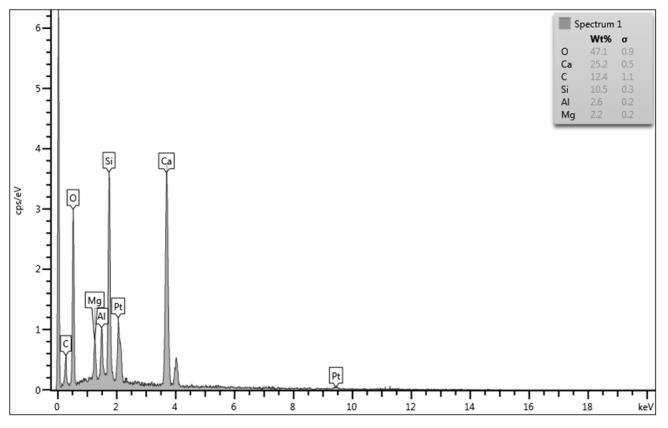
EDS graph of ferrochrome slag nanofiller.

Energy-Dispersive X-ray Spectroscopy (EDS) analysis was performed to obtain the qualitative composition of ferrochrome slag nanofiller. Various elements present in the nanofiller, with their peaks, are shown in the EDS graph (Figure 16). Table 5 provides the quantitative evaluation of the elements in weight percentage and atomic percentage.

### 3.3. XRD Analysis

**Figure 17:**
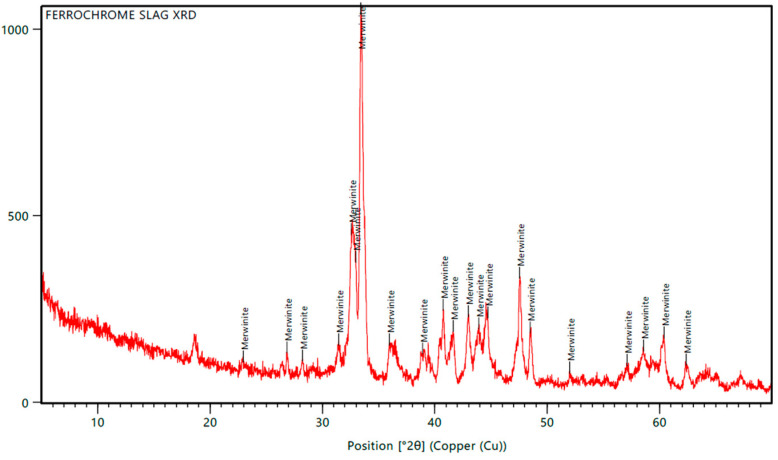
XRD spectrum of the composite with 2 wt.% nanofiller.

X-ray Diffraction (XRD) analyses were performed to examine the structural properties of ferrochrome slag, as shown in Figure 17. The results indicated that the merwinite (Ca_3_Mg_1_O_8_Si_2_) was the dominant crystalline phase present. Several diffraction peaks corresponding to merwinite were found at 18.6°, 22.97°, 26.85°, 28.21°, 31.44°, 32.57°, and 33.42°. The peak at 33.42° had the highest intensity, indicating significant crystallinity and high purity in the sample. The presence of merwinite was consistent in the data, showing notable peak intensities and distinct d-spacing values, which further confirmed the homogeneity of the slag’s mineral composition. These results indicate that the sample has a well-defined crystalline structure mainly composed of merwinite, which could affect its chemical reactivity and potential applications.

### 3.4. Fourier Transform Infrared Spectra (FTIR) Analysis

**Figure 18:**
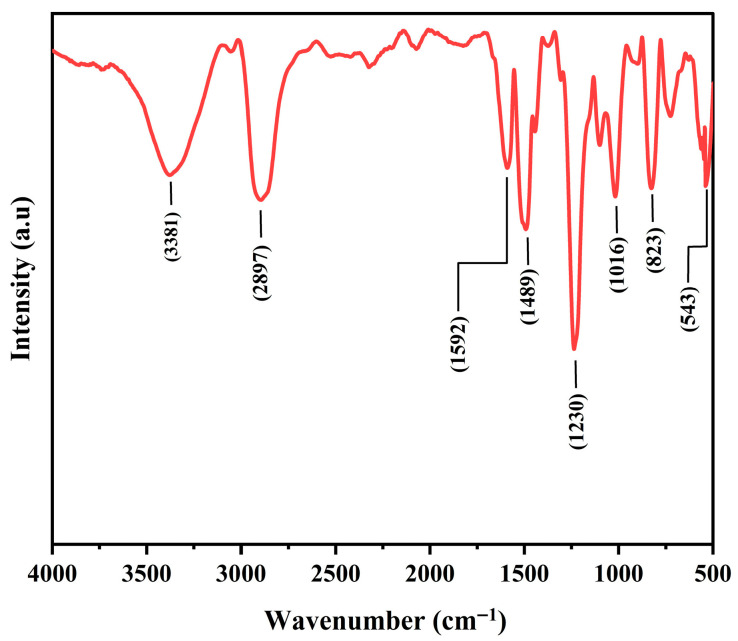
FTIR spectrum of the ferrochrome slag nanofiller.

FTIR analysis was performed to identify the various functional groups and molecular structure of the fabricated composite. The FTIR spectrum (Figure 18) of the composite with 2 wt.% of nanofiller loading displays characteristic absorption bands of the epoxy matrix, along with additional features arising from the slag filler. A broad band centered around 3400 cm^−1^ corresponds to O-H stretching vibrations, which is attributed to hydroxyl groups on the surface of the slag and adsorbed moisture. This indicates hydrogen-bonding interactions with the polymer network at 2 wt.% nanofiller. The peaks observed at 2925 and 2897 cm^−1^ are due to the C–H stretching in the epoxy backbone, while a weaker band near 1720 cm^−1^ is associated with carbonyl groups that may originate from residual curing agents or oxidative products. The band at 1592 cm^−1^ corresponds to aromatic C=C stretching in the epoxy resin, which overlaps with H-O-H bending from physically adsorbed water. Strong absorption bands in the range of 1230–1020 cm^−1^ are obtained from the superimposition of C-O-C stretching vibrations of the cured epoxy and Si-O-Si/Si-O-M (where M = Fe, Cr, Mg, Ca) stretching modes from the ferrochrome slag. This confirms the effective incorporation of the filler. The absence or significant reduction of the characteristic epoxide ring vibration at around 915 cm^−1^ indicates a high degree of cure in the composite. Moreover, distinct peaks in the 680–540 cm^−1^ range are assigned to metal–oxygen vibrations (Cr-O, Fe-O, Mg-O) that are characteristic of ferrochrome slag. These results verify the successful dispersion of ferrochrome slag within the epoxy matrix and suggest interfacial interactions between the polymer and the filler.

### 3.5. Tensile Strength

**Figure 19:**
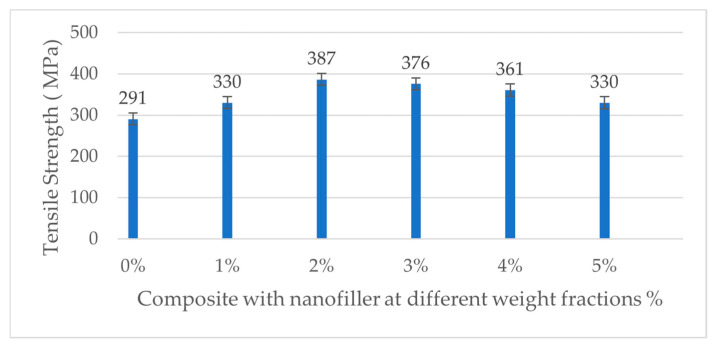
Tensile strength of neat and nanofilled composite at various weight fractions.

**Figure 20:**
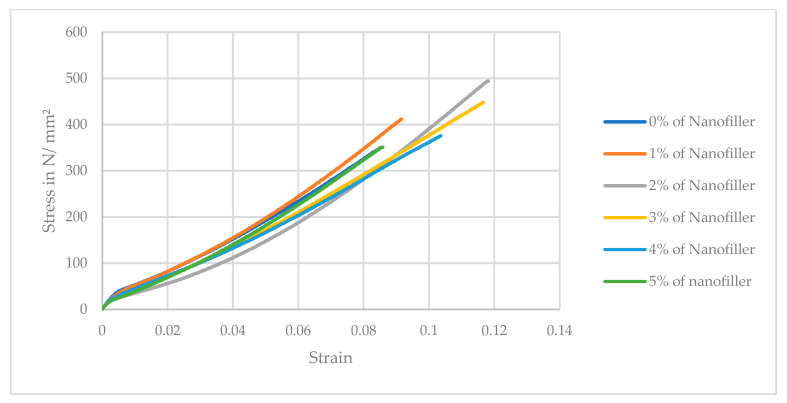
Stress–strain graph of the tensile test for neat and nanofilled composite at various weight fractions.

The tensile strength of the carbon fiber/epoxy composite shows a non-monotonic trend with increasing nanofiller content. As seen in Table 6, adding a small amount of ferrochrome slag nanofillers (1–2 wt.%) slightly increased the composite’s tensile strength compared to the unfilled composite. The neat composite (0% filler) had an average tensile strength of 290.7 MPa, which increased to 330.3 MPa at 1% and peaked at 386.7 MPa at 2% filler. This corresponds to about a 33.02% improvement in tensile strength at 2 wt.% nanofiller, suggesting that a low loading of well-dispersed rigid nanofillers can enhance load transfer in the matrix and potentially improve fiber–matrix interfacial stress distribution. However, beyond 2% nanofiller, the tensile strength declined distinctly. At 3% filler, the strength dropped to 375.8 MPa (falling below the neat value), and at 4% and 5% filler, it further reduced to 360.9 MPa and 330 MPa, roughly a 9.44% decrease relative to the unfilled composite. The error bars (standard deviation) indicate the downward trend at high filler loadings, as represented in Figure 19. These results demonstrate that while a small addition of ferrochrome slag nanopowder can marginally increase tensile strength, excessive filler (>3 wt.%) has a detrimental effect on the tensile performance, likely due to particle agglomeration and the introduction of defects (voids or weak interfaces) that facilitate earlier tensile failure. Therefore, a modest nanofiller addition (around 1–2 wt.%) provides a slight improvement in tensile strength, whereas higher loadings (3 wt.%) lead to substantial reductions below the strength of the unfilled composite. This trend can be explained by the interplay between particle reinforcement and damage mechanisms, where at low concentrations, the well-dispersed nanofillers can stiffen the epoxy matrix and improve the fiber–matrix interfacial shear transfer. Other reasons for improved strength include the rigid slag particles (which are rich in hard oxides) that act as micro-reinforcements and carry a portion of the load and constrain matrix deformation, thereby raising the composite’s resistance to initial microcracking [40]. Similar observations were reported in the literature, where adding micro/nanofillers up to an optimum percentage can increase a composite’s tensile and flexural strength due to enhanced load sharing and crack blunting effects [41,42]. On comparing with previous literature studies, ferrochrome slag nanofiller demonstrated better enhancement for tensile strength of 33.02%, exceeding ceramic fillers such as Al_2_O_3_ (29.54%) and SiC (25.27%) [2,19]. Furthermore, zinc oxide (ZnO) showed a moderate improvement of 24%, while the carbon nanotube provided the lowest improvement of tensile strength (10.4%) [20,22]. The stress–strain graphs are plotted as shown in Figure 20, which shows linear elastic behavior before failure. As shown in the bar graph, the highest tensile strength is observed for 2 wt.% nanofiller addition, after which the strength decreases.

### 3.6. Flexural Strength

**Figure 21:**
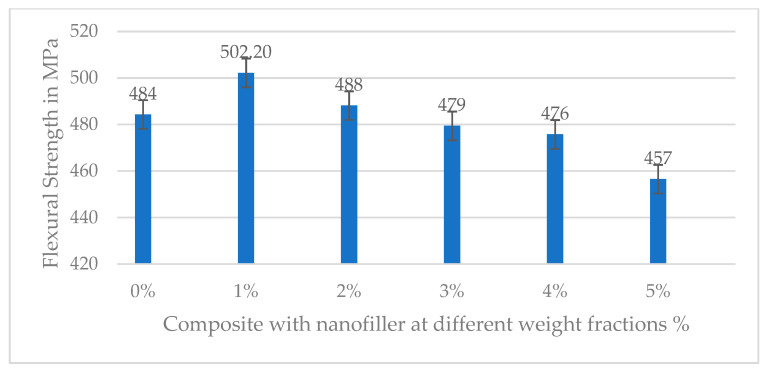
Flexural strength of neat and nanofilled composite at various weight fractions.

**Figure 22:**
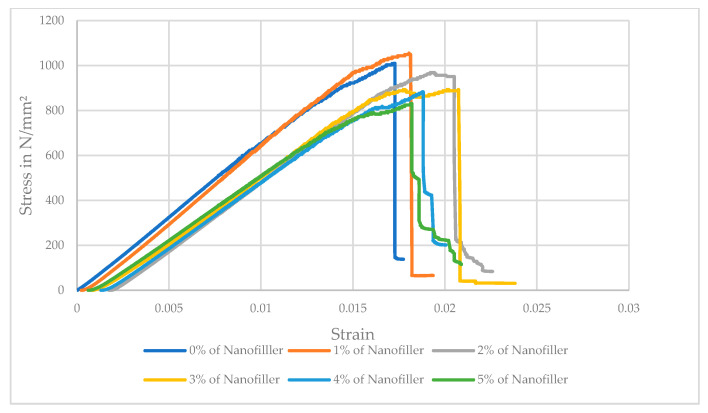
Stress–strain graph of the flexural test for the neat and nanofilled composite at various weight fractions.

The flexural strength exhibited a trend similar to the tensile strength, with an initial improvement at low nanofiller levels followed by a decline at higher contents. The baseline flexural strength for 0% filler was about 484.3 MPa, as shown in Table 7. Incorporating 1% slag nanofillers increased flexural strength to 502.2 MPa from 484.3 MPa. This peak represents a 3.7% increase over the unreinforced composite, indicating that a minor fraction of stiff filler in the matrix can reinforce the composite under bending loads. This improvement suggests the nanofillers helped stiffen the resin and possibly improved resistance to compression on the concave side of the flexural specimen. However, beyond 1% filler, the flexural strength progressively decreased. At 2% filler, the strength was 488.2 MPa (falling slightly below the neat value), and at 3, 4, and 5% nanofiller, the samples showed further reductions to 479.4, 475.8, and 456.5 MPa, as shown in the bar graph below (Figure 21). Thus, an optimal nanofiller content of around 1% yielded the maximum flexural strength. In contrast, higher loadings led to inferior flexural performance, presumably due to diminished resin ductility and stress concentrations around filler aggregates. Similar to tensile strength, after adding 2 wt.% of nanofiller, the flexural strength decreases due to the high nanofiller concentrations, as seen in the above SEM analysis, which tends to form agglomerates and increase the viscosity of the resin, making it difficult to achieve good wetting of the fibers and a void-free composite. The clustering of slag nanofillers creates localized stress concentrations and potential debonded regions in the matrix. Under bending loads, these defects become initiation sites for cracks, resulting in earlier failure at lower applied stress. This explains the sharp drop in flexural strength at 2–5% filler addition. As observed in the literature, researchers have noted that after a certain optimal filler loading, composites’ mechanical properties decline due to particle agglomeration, which introduces defects/voids [41,43]. Sathiya Narayanan et al. reported an 18–26% increase in the flexural strength of GFRP composites at 5 wt.% glass powder. However, further addition of the filler resulted in a decline in strength due to the fragility of the particles and their tendency to agglomerate, which induced micro-voids and increased brittleness. [41]. Our findings align with this behavior, though our optimum occurred at a slightly lower filler fraction (around 2% for tensile and 1% for flexural strength), likely due to differences in filler type and dispersion quality. Another factor contributing to improved strength is the effective dispersion of nanofillers. The combination of ultrasonication and mechanical stirring uniformly dispersed the nanofillers in the resin solution. These uniformly distributed nanofillers can efficiently carry the applied load, whereas agglomerated particles reduce the effective load-bearing area and act as potential crack initiators. Additionally, higher filler content means less epoxy resin is available to bind the fibers, potentially weakening the fiber–matrix interface. Stress–strain graphs are plotted for flexural strength, as shown in Figure 22. Initially, the curves represent linear elastic behavior, indicating good fiber and matrix bonding, along with nanofiller addition. As seen in the bar graph, the maximum flexural strength is noted for 1 wt.% nanofiller addition, and with the increasing filler content, the strength reduces.

### 3.7. Hardness Results

**Figure 23:**
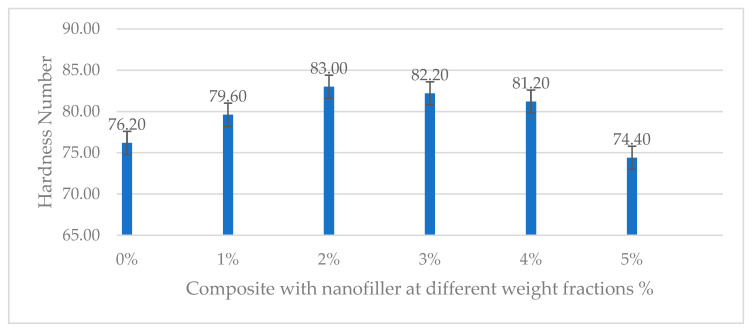
Hardness number of neat and nanofilled composite at various weight fractions.

Shore D hardness is relevant for fiber composites as it reflects the matrix–filler contribution to surface stiffness. The addition of ferrochrome slag nanofillers had a pronounced effect on the composite’s hardness. As shown in Table 8, the Shore D hardness increased steadily with filler content up to about 2 wt.% and then dropped off at the highest contents. The neat composite exhibited a hardness of 76.20 (Shore D). With just 1% nanofiller, the hardness rose to 79.60. The composite with 2% filler showed the highest hardness, 83 Shore D, representing a significant increase of 8.92% over the unfilled composite. This indicates that the dispersion of hard ferrochrome slag particles in the epoxy matrix effectively stiffened the surface and reduced the indentability of the material (the slag particles themselves are very hard and can raise the composite’s resistance to indentation). However, beyond this point, the hardness plummeted: the 3% nanofiller sample dropped to 82.20, and the 4% and 5% samples to 81.20 and 74.40, both falling slightly below the hardness of the 0% filler composite, as shown in Figure 23. This counter-intuitive drop at high filler loadings is likely related to poor particle distribution and the presence of micro-voids or resin-starved regions at excessive filler content—these defects can produce local soft spots or compliance, lowering the measured hardness. Overall, up to 2% nanofiller yielded a steadily increasing hardness due to the reinforcing effect of rigid particles, but beyond 2%, the composite’s hardness and surface uniformity deteriorated. The slag particles (rich in ceramic oxides) are significantly harder than the epoxy matrix; therefore, their presence restricts the indentation of the durometer pin [40]. This behavior is commonly observed where adding ceramic or mineral fillers raises a composite’s hardness due to the filler’s intrinsic hardness and the stiffening of the matrix–filler mixture. In the present work, the hardness climbed from 76.20 (neat) to 82.20 (2% filler), indicating a substantial improvement and demonstrating effective transfer of load to the hard particles during the indentation test. However, the expected drop in hardness at 3%, 4%, and 5% filler can be attributed to poor dispersion and microstructural inconsistencies at high filler content. If large agglomerates or void regions exist, the indenter might penetrate those softer spots more easily, yielding lower hardness readings.

### 3.8. Impact Strength

**Figure 24:**
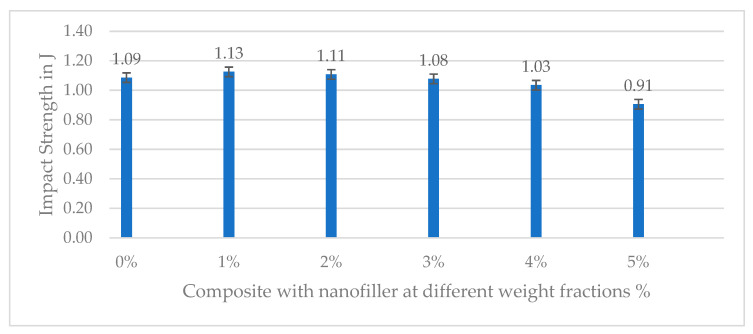
Impact strength of neat and nanofilled composite at various weight fractions.

The impact toughness (Charpy impact strength) as a function of nanofiller content showed a distinct optimum at moderate filler loading, with a sharp decline at the highest loadings. The unfilled CFRP composite had an impact strength of 1.09 J. At 1% filler, a notable increase in impact strength was observed, as shown in Table 9, reaching 1.13 J, which is approximately 3.62% higher than the 0% filler case. This suggests that at 1 wt.%, the presence of nanofillers has improved energy absorption through mechanisms like crack deflection, fiber–matrix debonding energy, or fiber pull-out that can dissipate impact energy. However, at filler contents beyond this optimum, impact resistance dropped significantly. The 2 wt.% composite had an impact strength of 1.11 J, which is lower than the neat composite, and at 3, 4, and 5% filler, it further decreased to 1.08, 1.03, and 0.91 J, as shown in Figure 24. Thus, the composites with 3–5% nano-slag were more brittle and had less impact energy than the neat composite. In conclusion, a moderate addition of nanofiller can slightly improve or maintain impact toughness, with 1% showing the best impact performance, whereas higher filler loadings cause the composite to become more notch-sensitive and brittle under impact loading.

## 4. Conclusions

In conclusion, the optimal ferrochrome slag nanofiller content lies in the range of 1–2 wt.%. At this range, the composite benefits from improved matrix properties and potentially enhanced fiber–matrix interaction without suffering from severe agglomeration or processing issues. The following conclusions were drawn from the above investigation:

1. Mechanical characterization revealed that the incorporation of slag nanofillers at low concentrations (specifically 1–2 wt.%) led to moderate improvements in tensile and flexural strength, while also enhancing impact resistance and surface hardness.
2. SEM analysis indicates uniform dispersion of nanofillers at low filler addition, whereas higher loading results in significant agglomeration and the presence of voids.
3. Optimal performance was achieved at 2 wt.% for tensile strength (386.7 MPa) and hardness (83), and at 1 wt.% for flexural strength (502.2 MPa) and impact energy absorption (1.13 J). These enhancements are attributed to improved stress transfer, matrix stiffening, and potential crack-arresting mechanisms introduced by the well-dispersed hard ceramic phases within the ferrochrome slag.
4. Beyond the optimum level (i.e., 1–2 wt.% filler addition), increasing the filler content above 1 wt.% for flexural and impact properties and above 2 wt.% for tensile and hardness resulted in a decline in performance. This reduction is due to poor dispersion, nanofiller agglomeration, and the formation of voids or weak interfaces that act as stress concentrators.
5. Ferrochrome slag nanofillers offer a promising route for enhancing epoxy-based CFRP systems at the optimum filler addition. This approach not only improves mechanical performance but also supports sustainable engineering practices by reducing industrial waste and promoting material reuse.

The present study highlights the environmental and economic advantages of utilizing industrial waste materials in high-performance composites. Transforming ferrochrome slag into a value-added nanofiller not only enhances composite performance but also advances sustainable material development and aligns with circular economy principles. Future research can focus on refining dispersion methods, implementing surface functionalization of slag particles, and assessing the long-term durability, wear resistance, and thermal behavior of the developed composites.

## Figures and Tables

**Table 1:** Types of fillers and their effect on mechanical properties.

Type of Filler	Mechanical Properties	Loading Range	Enhancement	References
Alumina (Al_2_O_3_)	Tensile Strength	1.75 wt.%	29.54%	[2]
	Flexural Strength		31.76%	
	Impact		47.51%	
	Hardness (HB)		47	
Silicon carbide (SiC)	Tensile Strength	1.25 wt.%	25.75%	[19]
	Flexural Strength		12.79%	
	Impact		30.45%	
	Hardness (HB)		43	
Carbon nanotube (CNT)	Tensile Strength	1 wt.%	10.4%	[20]
	Flexural Strength		41.86%	
	Impact		53.25%	
Nanoclay	Tensile Strength	2.50 wt.%	10.4%	[21]
	Flexural Strength		41.86%	
Zinc oxide (ZnO)	Tensile Strength	15 g	24%	[22]
	Flexural Strength		26%	
	Impact		18%	
	Hardness (HRC)		16	

**Table 2:** Sample composition.

Sample No.	Carbon Fiber Weight %	Epoxy Resin and Hardener Weight %	Nanofiller Weight %
1	50%	50%	0%
2	50%	49%	1%
3	50%	48%	2%
4	50%	47%	3%
5	50%	46%	4%
6	50%	45%	5%

**Table 3:** Ball milling parameters.

Total Time (min)	No of Milling Cycles	Speed (Rpm)	Ball Material	No of Milling Balls Used
105	7 cycles with 15 min on, 15 min off	400	Ceramics	Large-6 Medium-60 Small-600

**Table 4:** Particle size data of ferrochrome slag nanofiller from the SEM image.

Count	Mean (nm)	Standard Deviation	Minimum (nm)	Median (nm)	Maximum (nm)
20	291.85	46.83	5837	287	397

**Table 5:** EDS data of elements present in ferrochrome slag nanofiller with their atomic and weight percentages.

Element	Weight %	Atomic %
Calcium (Ca)	25.2	0.5
Silicon (Si)	10.5	0.3
Aluminum (Al)	2.6	0.2
Magnesium (Mg)	2.2	0.2
Oxygen (O)	47.1	0.9
Carbon (C)	12.4	1.1

**Table 6:** Tensile strength results at various filler loadings.

Composite	Tensile Strength (MPa)		Strength Gain %
	Average	Std. Dev.	
**Neat CFRP**	290.7	63.79	-
**1% of Nanofiller**	330.3	19.69	13.60
**2% of Nanofiller**	386.7	32.53	33.02
**3% of Nanofiller**	375.8	21.54	29.26
**4% of Nanofiller**	360.9	25.46	24.15
**5% of Nanofiller**	330.0	18.37	13.51

**Table 7:** Flexural strength results at various filler loadings.

Composite	Flexural Strength (MPa)		Strength Gain %
	Average	Std. Dev.	
**Neat CFRP**	484.3	6.36	-
**1% of Nanofiller**	502.2	10.32	3.70
**2% of Nanofiller**	488.2	17.47	0.79
**3% of Nanofiller**	479.4	5.94	−1.01
**4% of Nanofiller**	475.8	10.25	−1.77
**5% of Nanofiller**	456.5	12.02	−5.74

**Table 8:** Shore D hardness number at various filler loadings.

Composite	Hardness Number		Strength Gain %
	Average	Std. Dev.	
**Neat CFRP**	76.20	1.64	
**1% of Nanofiller**	79.60	5.68	4.46
**2% of Nanofiller**	83.00	3.87	8.92
**3% of Nanofiller**	82.20	6.26	7.87
**4% of Nanofiller**	81.20	9.13	6.56
**5% of Nanofiller**	74.40	1.48	−2.36

**Table 9:** Impact strength results at various filler loadings.

Composite	Impact Strength (J)		Strength Gain %
	Average	Std. Dev.	
**Neat CFRP**	1.09	0.05	-
**1% of Nanofiller**	1.13	0.02	3.62
**2% of Nanofiller**	1.11	0.08	2.03
**3% of Nanofiller**	1.08	0.07	−0.77
**4% of Nanofiller**	1.03	0.09	−4.70
**5% of Nanofiller**	0.91	0.08	−16.61

## Data Availability

The original contributions presented in this study are included in the article. Further inquiries can be directed to the corresponding authors.
